# Lysine-specific methyltransferase Set7/9 in stemness, differentiation, and development

**DOI:** 10.1186/s13062-024-00484-z

**Published:** 2024-05-29

**Authors:** Alexandra Daks, Sergey Parfenyev, Oleg Shuvalov, Olga Fedorova, Alexander Nazarov, Gerry Melino, Nickolai A. Barlev

**Affiliations:** 1 Institute of Cytology, Russian Academy of Sciences, St Petersburg, Russian Federation 194064; 2 Department of Experimental Medicine, TOR, University of Rome Tor Vergata, 00133 Rome, Italy; 3 Department of Biomedical Sciences, School of Medicine, Nazarbayev University, 001000 Astana, Kazakhstan

**Keywords:** Set7/9 methyltransferase, Histone modifications, p53, Differentiation, Stemness, Stem cells, Development, Embryogenesis, Cancer stem cells, iPSC

## Abstract

The enzymes performing protein post-translational modifications (PTMs) form a critical post-translational regulatory circuitry that orchestrates literally all cellular processes in the organism. In particular, the balance between cellular stemness and differentiation is crucial for the development of multicellular organisms. Importantly, the fine-tuning of this balance on the genetic level is largely mediated by specific PTMs of histones including lysine methylation. Lysine methylation is carried out by special enzymes (lysine methyltransferases) that transfer the methyl group from S-adenosyl-L-methionine to the lysine residues of protein substrates. Set7/9 is one of the exemplary protein methyltransferases that however, has not been fully studied yet. It was originally discovered as histone H3 lysine 4-specific methyltransferase, which later was shown to methylate a number of non-histone proteins that are crucial regulators of stemness and differentiation, including p53, pRb, YAP, DNMT1, SOX2, FOXO3, and others. In this review we summarize the information available to date on the role of Set7/9 in cellular differentiation and tissue development during embryogenesis and in adult organisms. Finally, we highlight and discuss the role of Set7/9 in pathological processes associated with aberrant cellular differentiation and self-renewal, including the formation of cancer stem cells.

## Background

Post-translation modification (PTM) of proteins is one of the key mechanisms regulating vital cellular functions. Along with acetylation, phosphorylation, ubiquitination, and SUMOylation, methylation is one of the major classes of PTMs. Methylation of lysine and arginine residues is performed by lysine- and arginine-specific methyltransferases (KMTs and PRMTs), respectively. Both groups of enzymes use S-adenosyl-L-methionine (SAM) as a donor of the methyl group. Mono- and di-methylation variants exist for both lysines and arginines, while only lysines can be in a trimethylated state [1, 2].

The main families of KMTs are SET (Su(var) 3-9,E(z),Trithorax)- and DOTL-1 (DOT1-like)- domain containing KMTs. All organisms, including procaryotes, fungi, and plants, were shown to have SET domain-containing proteins. This evolutionary conservative domain is responsible for the methylation of protein substrates, providing the binding of methyl group donor SAM with targeted lysine residue. Importantly, SET domain-containing proteins perform histone methylation function in all groups of organisms throughout the evolution [3–5].

**Fig. 1:**
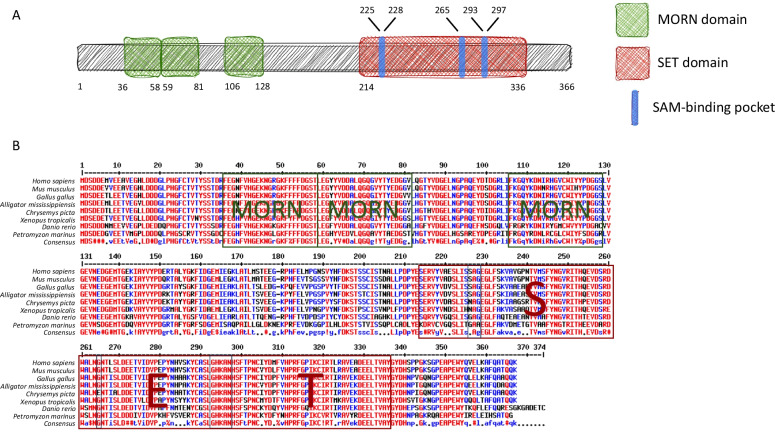
**A** The domain organization of Set7/9 protein. **B** The alignment of Set7/9 amino acid sequences of selected species. Amino acid numbering is according to *Homo sapiens*

SET domain-containing methyltransferase Set7/9 (alternatively named Setd7, Set7, Set9, and KMT5) is coded by the SETD7 gene. Set7/9 expressed in the human cervical cancer cell line HeLa was purified biochemically and characterized in Zhang’s and Reinberg’s labs almost simultaneously in 2001 [5, 6]. The structure of the human Set7/9 protein includes 366 amino acids comprising three MORN (Membrane Occupation and Recognition Nexus) motifs responsible for protein–protein interactions in the N-terminal part and the SET domain in its C-terminus (Fig. 1).

Evolutionarily, Set7/9 first appeared in fish and was characterized by a high degree of conservation among different species [7]. Indeed, analysis of the latest versions of genome assemblies allowed us to reveal the Set7/9-coding gene in Agnatha (Jawless fish). The alignment of amino acid sequences of Set7/9 proteins [5, 6] from *Homo sapiens* (Mammalia, Primates), *Mus musculus* (Mammalia, Rodents), *Gallus gallus* (Birds), *Alligator mississippiensis* (Reptilia, Crocodiles), *Chrysemys picta* (Reptilia, Turtles), *Xenopus tropicalis* (Amphibia), *Danio rerio* (Bony fish), and *Petromyzon marinus* (Jawless fish) revealed a high level of homology at the protein level between these evolutionary distant groups (Fig. 1). The fact that this homology extends beyond its catalytic SET domain strongly supports the idea that Set7/9 likely exerts the same function(s) in these species. It is worth noting that such a high homology distinguishes Set7/9 from other SET domain-containing KMTs, for example, EZH2.

Set7/9 was first described as H3K4-specific histone methyltransferase. However, further studies revealed many additional targets of Set7/9—both histones and non-histone proteins—are participating literally in all major cellular processes [8–12]. This review is focused on the role of Set7/9 in cell differentiation and the development of organs and tissues in different organisms during embryogenesis. The importance of Set7/9 in the determination of stem cells (CSs) fate as well as in cancer stem cells (CSCs) formation is also discussed.

## The role of Set7/9 in the histone modifications landscape

One of the most distinguishing characteristics of SCs is their bi-valent state of histone modifications that provide SCs a remarkable transcriptional plasticity, which allow SCs to respond swiftly to various signaling cues. Chromatin PTMs endure substantial changes throughout differentiation and development. Histone methylation is essential for all developmental stages and the maintenance of pluripotency. Methyl groups attached to lysines or arginines of canonical histones and their variants significantly affect the chromatin structure and, therefore, gene expression.

**Fig. 2:**
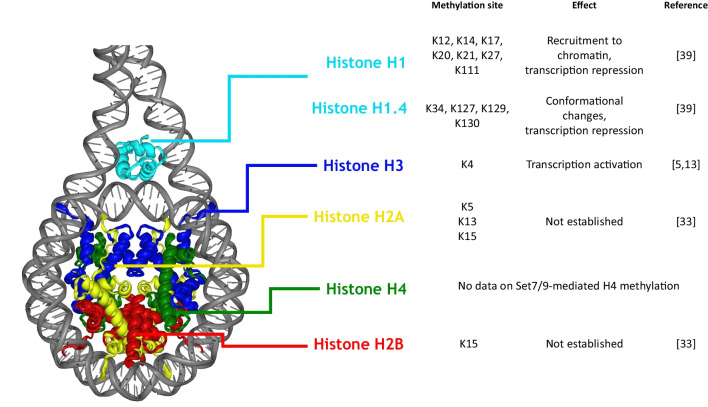
The participation of Set7/9 in histone methylation

Originally, Set7/9 protein was described as performing monomethylation of lysine 4 of histone H3 (H3-K4Me1), thus activating transcription [5, 13] (Fig. 2). Additionally, Set7/9-induced methylation of H3 at K4 prevents the repressive methylation of H3 at the near lysine residue K9, which is mediated by Suv39h1and other H3-K9-specific HMTases [14]. Since H3K9 methylation is considered to be a repressive mark, Set7/9-mediated suppression of this methylation event apparently contributes to activation of gene expression [5].

Indeed, the methylated form of histone H3 at lysine 4 is one of the key marks of the actively-transcribed chromatin, since H3K4 methylation promotes subsequent acetylation of H3 leading to transcription activation [15, 16]. For example, in human embryonic stem cells (hESC) the level of methylated H3K4 is enriched with the H3K4Me3 mark at promoters of many genes whose products signify the pluripotent state of hESCs [17, 18]. On the contrary, global levels of H3K4Me3 decrease in differentiation conditions (for example, upon retinoic acid-induced differentiation of murine ESC (mESC) [19] indicating the key role of this modification in the induction and maintenance of stem cell identity.

The histone profile of SC is also characterized by the presence of bivalent chromatin domains around transcription initiation sites of the key differentiation genes (such as Polycomb, Trithorax and HOX genes) [20]. These bivalent domains combine both transcriptionally “active” H3K4Me3 and “silent” H3K27Me3 (or H3K9Me in mesenchymal stem cells (MSCs)) marks, providing the plasticity to SCs by keeping them in the undifferentiated state, yet poised to differentiation [21, 22]. Upon differentiation, the promoters of these genes lose H3K27Me3 enrichment against maintaining high levels of H3K4Me3 which is the stimulus to initiate the expression [23].

That lysine methylation is a processive modification (monomethylation can be converted into di- and tri-methylation) hints that Set7/9-mediated methylation may be a priming event for subsequent di- and trimethylation mediated by the COMPASS complex [24–28]. This assumption is also supported by the data on the association of Set7/9 expression with elevated dimethylation of the promoter region of the insulin gene (INS) in pancreatic β cells [29].

Furthermore, the distribution of the H3K4 monomethylation mark in the regulatory regions of genes differs from that mapped for di-and trimethylated lysine variants [30, 31]. Monomethylation was found to cover wider areas around transcription start sites in comparison to H3K4Me2 and H3K4Me3, and, importantly, it localizes mainly in the area of enhancers of actively expressing genes [13, 32]. Taken together, H3K4 methylation seems to be one of the critical mechanisms by which Set7/9 regulates the process of gene expression during the SCs differentiation process.

In vitro studies also demonstrated the ability of Set7/9 to methylate histone H2A at K5, K13, K15 and H2B at K15 sites [33] (Fig. 2). However, the role of these Set7/9-driven modifications of H2A and H2B remains unclear. It was repeatedly shown that H2A histone undergoes DNA damage-induced ubiquitination at K13 and K15 by E3 ligase RNF168 [34, 35]. Possibly, Set7/9- and RNF168-derived modifications may counteract each other, but this assumption and the role of H2A and H2B methylation in differentiation and development warrants further investigation.

The fact that methylation of H2A and H2B was not detected in the histone octamer [33] and that Set7/9 has no methyltransferase activity toward the nucleosome-incorporated histone H3 [11, 36, 37] indicate that Set7/9 may act during DNA replication only on free histones H2A, H2B, and H3 before their incorporation into the nucleosome [33, 38].

Linker histone H1 is another *bona fide* target of Set7/9-mediated methylation. It was found that Set7/9 methylates H1.0 at lysine residues K12, K14, K17, K20, K21, K27, K111, and H1.4 at K34, K127, K129, and K130 sites in human-derived induced pluripotent stem cells (iPSCs) and these modifications change the conformation of H1 [39] (Fig. 2). Additionally, knockdown of Set7/9 correlated with a decreased recruitment of H1 to the promoters of Nanog and OCT4 genes in iPSC subjected to in vitro differentiation [39]. Indeed, the elevated expression of H1 and its inclusion in chromatin have a suppressive effect on transcription. The suppressive function of H1 variants depends significantly on PTMs and is largely manifested in the differentiation process: SCs employ this mechanism to inhibit the expression of genes responsible for pluripotency [40].

Collectively, the data presented so far point to the fact that Set7/9 is an active participant of the “histone code” writing process and in doing so, Set7/9 controls the balance between the pluripotent and differentiated states of cells.

## Stemness-associated non-histone targets of Set7/9

### Set7/9-mediated regulation of Suv39h1

**Fig. 3:**
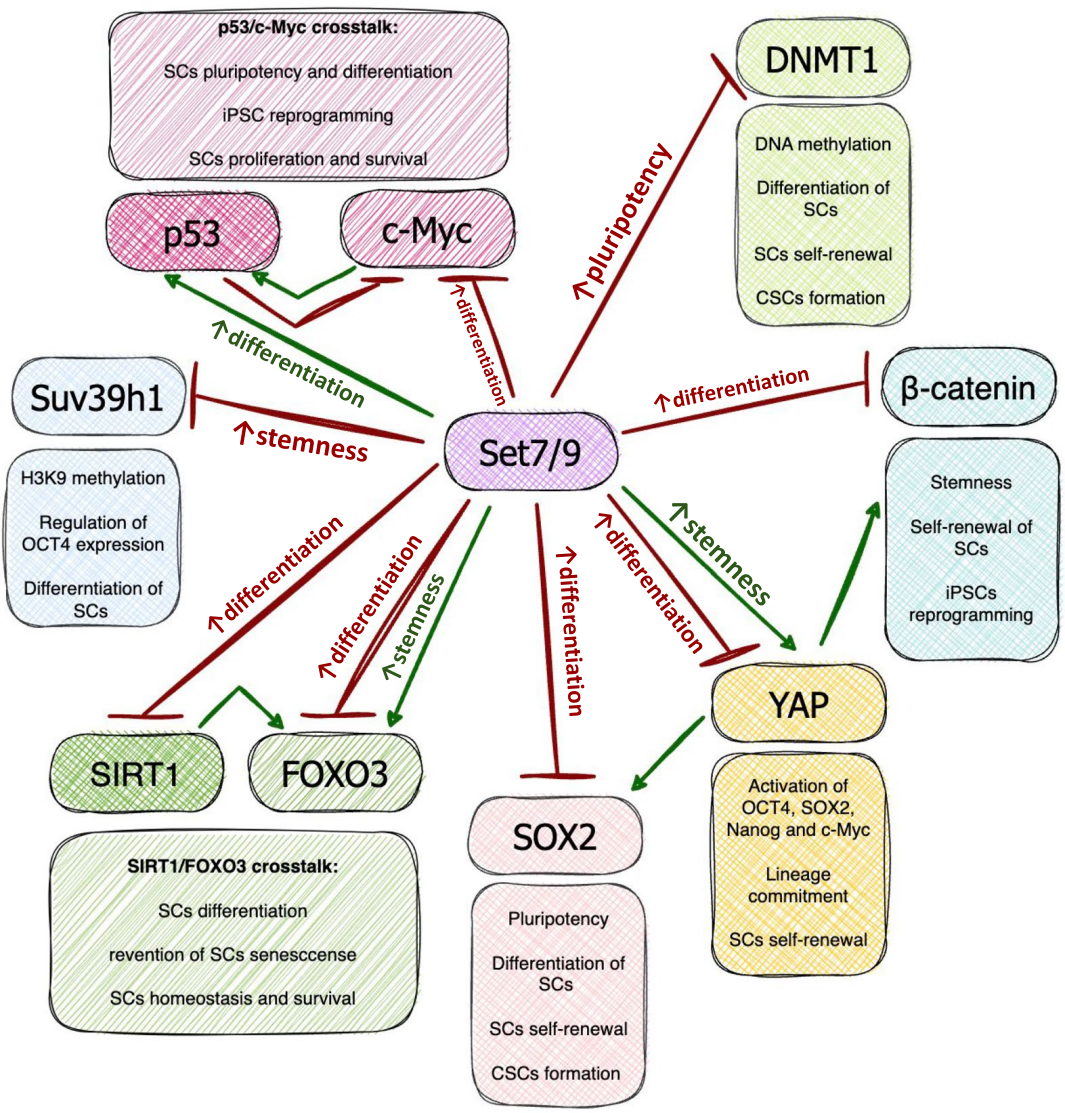
The scheme illustrating the participation of Set7/9 in regulation stemness/differentiation-related factors and pathways

The possible interaction of Set7/9 and Suv39h1 that was discussed above in the context of interplay between H3K4 methylation and H3K9 methylation can be further extended to their physical interaction on the protein level. Set7/9 has been shown to methylate directly lysines 105 and 123 of Suv39h1, resulting in downregulation of its methyltransferase activity and consequently in heterochromatin relaxation [41] (Fig. 3).

Suv39h1 is the protein methyltransferase (PKMT) which exerts trimethylation of histone H3 at lysine 9 and plays an important role in forming bivalent chromatin domains in stem cells [42] (Fig. 3). For example, Suv39h1 performs H3K9Me3 methylation that ignites the HP1a deposition at the *Oct4*-coding gene hence causing OCT4 suppression. Mechanistically, it was demonstrated that pseudogene-coded Oc4P4 lncRNA is upregulated upon differentiation of murine ESC (mESC) and recruits Suv39h1 to the *Oct4* promoter [43].

Another study reports that expression of Suv39H1 (and its homolog Suv39h2) as well as di- and trimethylation of H3K9 was the highest at early stages of neurogenesis and decreased during differentiation in mouse adult hippocampal progenitors (AHP) [44]. Additionally, the suppression of Suv39h1 via specific shRNA-mediated knockdown or using a chaetocin inhibitor resulted in impaired neuronal differentiation of progenitor cells [44]. This fact suggests that, acting as Suv39h1 antagonist, Set7/9 may promote stem cell properties in both mouse and mESC models as described in these studies. This observation additionally highlights the ability of Set7/9 to promote gene expression through direct or indirect H3K4 methylation.

Finally, Suv39h1 affect the terminal differentiation of CD8^+^ T lymphocytes, at least in mice. Thus, Suv39h1 silences stem/memory genes in short-living effector T cells, while T cells with Suv39h1 knockout demonstrate high expression of lymphoid stem cell markers, prolonged survival and increased long-term memory and reprogramming capacity [45]. Following the same concept of Set7/9 being a suppressor of Suv39h1, one might hypothesize that Set7/9 via Suv39h1 also contributes to the maintenance of the population of long-lasting memory T lymphocytes. However, this possibility needs further experimental validation.

### DNMT1 as a target of Set7/9

DNA-methyltransferase 1 (DNMT1) is the key genome methyltransferase in mammals that performs DNA methylation and maintains the patterns of CpG methylation in proliferating cells. DNMT1 plays an important role in the maintenance of adult SCs. Accordingly, the normal functioning of DNMT1 is essential for self-renewal of mesenchymal [46] and hematopoietic SCs [47], maintenance of undifferentiated state of neuronal SCs and appropriate hippocampus development [48, 49]. This enzyme is also required for retention of the proliferative potential and suppression of differentiation of epidermal progenitor cells [50].

Given the critical role of DNMT1 in genome stability, cell fate determination, and mammalian development it was expected that the homozygous inactivating mutation in the *Dnmt1* gene would cause embryonic lethality in mice [51]. Contrary to these expectations, it was demonstrated that knockout of the *Dnmt1* gene in mESC did not interfere with the ability to self-renew but instead impaired the differentiation process [51]. Another study demonstrated that downregulation of this methyltransferase facilitated murine cells reprogramming to a pluripotent state [18] (Fig. 3).

In 2009, two research groups independently demonstrated the ability of Set7/9 to downregulate DNMT1 (Fig. 3). First, Wang et al. showed that Set7/9 methylates DNMT1 at K1096 in vitro, which in turn, negatively regulates the protein level of DNMT1. On the contrary, demethylase LSD1 removes this methyl mark in vitro and stabilizes DNMT1 in murine ES cells [52]. A few months later, the Sriharsa Pradhan research group confirmed the direct Set7/9-DNMT1 interaction and their intranuclear colocalization in COS-7 fibroblast-like monkey cells. Furthermore, Set7/9 was shown to monomethylate DNMT1 at K142 resulting in the proteasome-dependent degradation of DNMT1 in human HeLa cells [53]. This mechanism is reminiscent of Set7/9-mediated regulation of HIF1a stability [54]: methylation of HIF1a by Set7/9 caused ubiquitination of the former and its subsequent proteasomal degradation [9, 54, 55]. Thus, Set7/9 methylates DNMT1 at least at two sites (K142 and K1096) and acts as a negative DNMT1 regulator in different cell types, including SCs.

### SOX2 as substrate of Set7/9

SOX2 is one of the key stemness factors that, in line with OCT3/4 and Nanog provides SC pluripotency maintenance and self-renewal [56–60]. The study by Jiemin Wong demonstrated the ability of Set7/9 to methylate SOX2 at K119 site in murine ESC [61]. Set7/9-mediated methylation led to subsequent ubiquitination of SOX2 by WWP2 E3 ligase and proteasomal degradation of SOX2 [61]. Interestingly, AKT-mediated phosphorylation of SOX2 at T118 counteracts Set7/9-mediated methylation and stabilizes SOX2 in ESC. Here, the negative effect of Set7/9 on SOX2 transcriptional activity and the importance of Set7/9 in appropriate cellular differentiation during embryogenesis was shown. A more recent study confirms the ability of Set7/9 to methylate SOX2 in human ovarian teratocarcinoma cells PA-1 at K117 (analogous to K119 in murine SOX2) [62]. The K42 site of SOX2 was also shown to be targeted by Set7/9-mediated methylation in this study. Importantly, similar to DNMT1, methylation at both K42 and K119 lysins promotes SOX2 proteasomal degradation [62]. LSD1 in turn was shown to demethylate and promote SOX2 stabilization both in PA-1 and mESCs, counteracting the Set7/9 pro-differentiation function [62].

### The role of Set7/9 in Wnt/β-catenin and Hippo/YAP signaling pathways

Wnt/β-catenin signaling is considered to be one of the important pathways supporting stemness, SC self-renewal, and pluripotency, as well as participating in iPCS reprogramming (reviewed in [63–67]) (Fig. 3). The Hippo signaling pathway participates both in stem state maintenance and cell lineage fate determination [68]. Under Hippo signaling activation through MST1/2 phosphorylation, YAP and TAZ proteins, being the main effectors of this cascade, are retained in the cytoplasm in the complex with 14-3-3 and targeted for proteasomal degradation. Oppositely, when Hippo is attenuated, YAP/TAZ translocate to the nucleus and activate the expression of such pluripotency promoting genes as OCT4, SOX2, Nanog, and c-Myc [69–72] (Fig. 3).

Set7/9 acts as a methyltransferase for both β-catenin and YAP (Fig. 3). In 2015, Shen et al. demonstrated that Set7/9 interacts with and methylates β-catenin at K180 in vitro and in vivo, and this modification results in an increase in GSK3β-mediated β-catenin degradation and suppression of WNT target genes. This process was shown to be facilitated in human cancer cells in response to oxidative stress [9, 73]. The recent study confirmed the significant role of Set7/9-mediated methylation of β-catenin at K180 in the generation of hematopoietic progenitor cells (HPCs) from hESC. That Set7/9 ablation results in stabilization of β-catenin and, consequently, WNT signaling activation, promotes paraxial mesoderm formation and the generation of hematopoietic and endothelial progenitor cells [74]. Therefore, this study highlighted Set7/9 participation in the determination of hESC differentiation towards the lateral plate or paraxial mesoderm and, accordingly, the formation of corresponding derivatives.

Numerous studies demonstrated the role of Set7/9 in YAP downregulation. In 2013, Oudhoff et al. revealed that mice lacking Set7/9 were characterized by a larger population of intestine progenitor cells, elevated nuclear accumulation of YAP, and upregulated YAP target genes, Ctgf and Gli20 that participate in the differentiation process [75]. In this study, Set7/9-mediated YAP K494 monomethylation was shown to promote YAP cytoplasmic retention and downregulation of YAP transcription targets [75]. Later, the same research group supported these findings by using a small molecule inhibitor of Set7/9 methyltransferase activity, (R)-PFI-2. Accordingly, (R)-PFI-2 treatment of mouse embryonic fibroblasts (MEFs) and human BC MCF7 cell lines enhanced the stability and nuclear localization of YAP, as well as augmented the expression of YAP1 transcriptional targets [76]. Another study demonstrated that Set7/9 may additionally inhibit YAP indirectly by promoting the activity of the YAP negative regulator, Hippo pathway transducer protein, LATS1 [77].

The study of Francesco Paneni group has suggested that Set7/9-mediated suppression of YAP through its methylation played role in myocardial ischemia and myocardial infarction caused by glucose deprivation in vivo. Thus, Set7/9 knock out mice demonstrated decreased infarct area and preserved the cardiac contractile function in the myocardial ischemia and myocardial I/R injury mouse model [78, 79]. Apparently, these effects were associated with the loss of Set7/9-mediated YAP methylation which otherwise traps the transcriptional co-activator YAP1 in the cytoplasm and prevents transcription of its antioxidative target genes, catalase (*Cat*) and superoxide dismutase (*Sod*) [78, 79]. Accordingly, ablation of Set7/9 re-activates the anti-oxidative functions of YAP1 in the myocardium.

Additionally, a recent study revealed the significance of YAP downregulation by Set7/9 in liver cancer progression. Knockout of the autophagy factor SPTBN1 in hepatic stem cells (HSCs) caused Set7/9 decrease, followed by subsequent YAP stabilization and nuclear translocation. Since YAP1 plays a pro-proliferative function in epithelial cells, the latter event promoted the onset of hepatocellular carcinoma (HCC) [80].

Another interesting study demonstrating the existence of the Set7/9-β-catenin-YAP signaling circuit in intestines was performed by Oudhoff and colleagues in 2016 (Fig. 3). Thus, it was shown that mice with targeted depletion of Set7/9 in intestinal epithelial cells exhibited a reduced level of carcinogenesis in the large intestine caused by dextran sodium sulfate [81]. Set7/9 was shown to be a component of the protein regulatory complex containing β-catenin, YAP, and Axin1. Mechanistically, Set7/9-mediated YAP1 methylation augmented the Wnt-induced nuclear accumulation of β-catenin and activation of several stemness and tumorigenesis factors including Lgr5, Olfm4, and Axin2 [81].

Taken together, the data on Set7/9 participation in regulation of both Wnt/β-catenin and Hippo/YAP signaling pathways strongly indicate the crucial role of this methyltransferase in the orchestration of stemness and self-renewal.

### The FOXO3/SIRT1 *axis* and Set7/9

Both Forkhead box O3a (FOXO3) longevity-associated TF and a member of the NAD-dependent family of sirtuin deacetylases SIRT1 are known to play instrumental roles in stem cells homeostasis, self-renewal, and differentiation [82–90]. Importantly, these factors are tightly connected in the context of stem state regulation (Fig. 3).

In line with this notion, the role of SIRT1/FOXO3 crosstalk in osteogenesis was demonstrated by the Dengshun Miao research group. According to the results demonstrated by this group, an overexpression of SIRT1 in murine MSCs lead to stimulation of the bone marrow MSCs differentiation into osteoblasts [91]. Mechanistically, the SIRT1-mediated deacetylation of FOXO3 led to an elevation of FOXO3 itself and its transcriptional target, anti-oxidative gene, *Sod2*. The latter event resulted in ROS neutralization, oxidative stress reduction, stimulation of osteoblasts formation, and prevention of MSCs senescence [91].

The role of Set7/9 in regulation of FOXO3 was also studied in the context of the oxidative stress response. For example, Xie et al. demonstrated that Set7/9-mediated FOXO3 methylation at K270 suppressed the transactivation activity of FOXO3 towards the pro-apoptotic *Bim* gene [92]. As a consequence, decreased levels of apoptosis in rat cerebellar granule neurons were detected in response to hydrogen peroxide treatment [92] (Fig. 3).

Another study demonstrated that Set7/9 methylates FOXO3 at lysine 271. Interestingly, the same lysine undergoes deacetylation mediated by SIRT1. Importantly, unlike acetylation, Set7/9-methylation of FOXO3 at K271 was shown to modestly activate its transcriptional activity in human HEK293T cells [93] (Fig. 3).

Strikingly, Set7/9 also methylates SIRT1 at four sites: K233, K235, K236, and K238 [94]. Set7/9-mediated methylation of SIRT1 caused a significant reduction of SIRT1 deacetylase activity towards p53 in HCT116 human colon cancer cells under genotoxic conditions. In turn, this event promoted p53 stabilization and activation of p53 target genes [94]. Thus, the participation of Set7/9 in regulation of SIRT1 and FOXO3 activities strongly hints towards the existence of such regulatory loop in stem cells. Again, this hypothesis requires additional experimental validation.

### c-Myc-p53-Set7/9 crosstalk

The proto-oncogene transcription factor c-Myc plays a paramount role in the biology of stem cells. Along with OCT3/4, SOX2, and Klf4, c-Myc is a component of the “Yamanaka cocktail” of transcription factors used for generation of iPSC [95]. Importantly, c-Myc itself participates in both SC pluripotency maintenance, self-renewal, and differentiation towards certain lineages [96–100] (Fig. 3).

The major molecular actor that counteracts the c-Myc functioning in SCs is the p53 human tumor suppressor. It was shown that murine MSCs obtained from the partially p53-inactivated mice are characterized by the upregulation of c-Myc. This phenotype is associated with increased proliferation, genomic instability, and a lack of senescence features [101] (Fig. 3). Negative regulation of c-Myc by p53 was also demonstrated in neuronal stem cells (NSCs) obtained from p53 knockout mice. Depletion of p53 increased the level of c-Myc expression as well as the proliferation rate of NSCs, and inhibited the differentiation potential [102].

p53-mediated suppression of c-Myc also affected its ability to drive hepatic CSCs renewal [103]. In this study, c-Myc-dependent activation of NANOG, OCT4, and EpCAM in hepatic cancer cells was also observed [103]. Interestingly, the dose-dependent switching of c-Myc functions was shown: from stemness-promoting while c-Myc is expressing at moderate levels to pro-apoptotic when c-Myc exceeds a threshold expression level [103].

Set7/9 is known to regulate p53 directly through methylation of p53 at the 372 lysine residue, and this modification was shown to contribute to p53 stabilization, its nuclear translocation, and consequently augmented transactivation of p53 target genes [8] (Fig. 3). It is fair to assume that the same modification also aids to p53-dependent repression of c-Myc. However, along with p53 positive regulation, Set7/9 was shown to interact with and stabilize the main p53 negative regulator, Mdm2, under genotoxic conditions [104]. Moreover, our research group recently demonstrated that Set7/9 acts as a suppressor of metabolic functions of c-Myc thereby participating in the regulation of glycolysis in lung cancer cells [105]. Given that glycolysis is critical for the maintenance of stem cells [106] and Set7/9 attenuates the expression of c-Myc, the key glycolytic transcription factor, makes it plausible that Set7/9 regulates stem cells via controlling the p53-c-Myc crosstalk (Fig. 3).

In addition to the protein targets described above, Set7/9 was shown to methylate such proteins as HIF1a, p65/RelA, E2F1, PARP1, and others (reviewed in [10, 107, 108]), each of which participates in different aspects of CSs vitality and functioning [109–113]. Indeed, Set7/9 is an important player in stemness and differentiation regulation, and its role in these processes will be discussed below.

## Set7/9 as a regulator of stemness, differentiation and development

### The role of Set7/9 in ESCs and iPSC differentiation

**Fig. 4:**
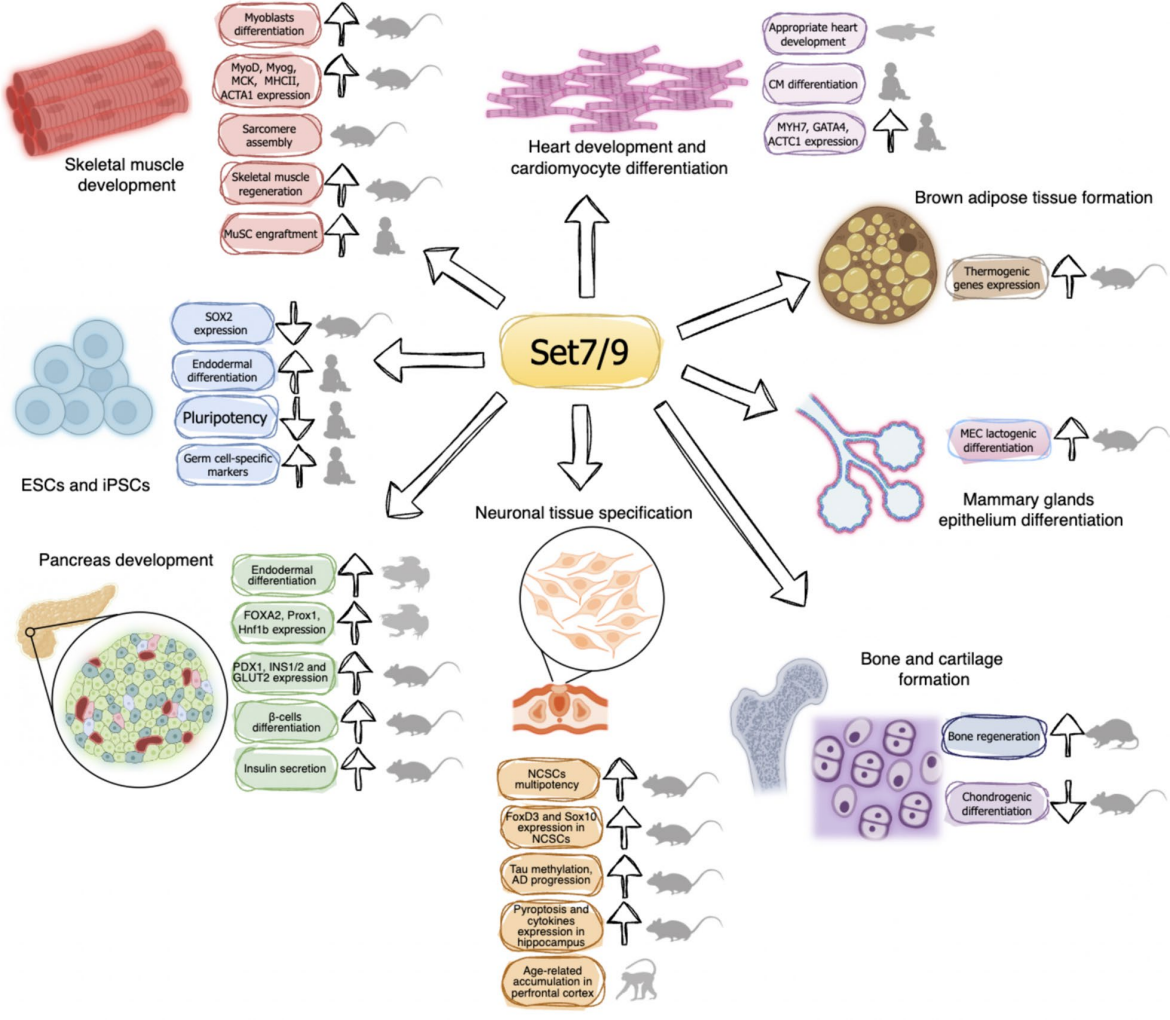
The role of Set7/9 in cell differentiation, organogenesis and different tissue types formation. The gray symbols indicate the model object or cell line source (human, rhesus macaque, mouse, rat, Xenopus)

As it was discussed above, Set7/9-mediated methylation of SOX2 attenuated its level in mESC [61]. Moreover, Set7/9 knockdown led to attenuated retinoic acid-induced differentiation of ESCs, promoting the population of SOX2-positive undifferentiated cells [61] (Fig. 4). In concordance with the differentiation-promoting role of Set7/9 is the finding that in hESC Set7/9 is expressed at the low level but its expression increases during the transit from pluripotent to multipotent state [114] (Fig. 4).

In contrast to previously described studies, Kim et al. reported a stemness-promoting role of Set7/9 in hESCs [115]. The authors showed that Set7/9 to methylates and stabilizes the LIN28A protein. The latter event results in the nuclear translocation and sequestration of pri-let-7 microRNA in hESCs [115] thereby preventing its maturation. Since one of the targets of -let-7 is c-Myc, the Set7/9-mediated attenuation of let-7 miR maturation would result in stabilization of c-Myc expression and promotion of pluripotency [115]. Undoubtedly, participation of Set7/9 in microRNA regulation in SCs [116–118] needs to be further investigated.

A key step for the successful reprogramming of mesenchymal fibroblasts into iPSCs is mesenchymal-epithelial transition (MET). MET is opposite to Epithelial-Mesenchymal Transition (EMT), and together they form the phenotypic plasticity of cells [119]. It is important to note that one of the main targets of Set7/9, the tumor suppressor p53, plays an important role in both processes. For example, the expression of Zeb1, one of the key factors in EMT induction, is suppressed by p53 due to the activation of p53-dependent microRNA-200c [120]. On the contrary, targeted knockdown of p53 expression attenuates the miR-200c expression and activates the EMT program, which is accompanied by an increase in the mammary stem cell population [121, 122].

In this regard, Montenegro et al. [123] showed that Set7/9 inhibits EMT by increasing the expression level of E-cadherin and decreasing the levels of the mesenchymal proteins vimentin and epidermal growth factor receptor (EGFR). Moreover, in MCF-7 epithelial cells, targeted downregulation of Set7/9 increased the population of cancer stem-like cells (CD44+/CD24−/low) and dedifferentiation of mammospheres, which was associated with a decrease in E-cadherin expression. At the molecular level, Set7/9 has been proposed to control CDH1 expression through enrichment of H3K4me and suppression of DNMT1 levels in cells [53, 124].

It is interesting to note that although the p53 tumor suppressor gene acts as a limiter on the phenotypic plasticity of cells, other members of its family, p63 and p73 [125], play the opposite role. Both p63 and p73 act as stimulating factors for efficient reprogramming by Yamanaka factors [119]. Accordingly, iPSCs derived from p73-deficient cells have a defective epithelial phenotype and changes in the expression of pluripotency markers. In this regard, it would be interesting to see the effect of Set7/9 on the transcriptional activity of p73 in reprogrammed cells.

An interesting study was performed on both hESC and human iPSCs. Again, Set7/9 was shown to express at a low level at the blastocyst stage and to increase during differentiation [126]. Notably, Set7/9 morpholino (MO) transfected (with and without overexpression of DNMT3B DNA de novo methyltransferase) human adult fibroblasts formed colonies resembling iPSCs and were characterized by the expression of pluripotency markers and suppressed proliferation rate. One more important observation made in this study is that the obtained DNMT3B-GFP/SETD7-MO transfected colonies displayed the expression of such germ cell-specific markers as Pumilio Homologs 1 and 2 (PUM1/2) and STELLAR, which suggests the role of Set7/9 in germ line identity during organism development [126].

Based on the foregoing, it is likely that Set7/9 plays critical role in switching of cellular program from pluripotency to differentiation during mammalian development (Fig. 4). Indeed, the impact of Set7/9 in particular tissue formation will be summarized below.

### Set7/9 as a regulator of cardiac development

Several studies demonstrate the crucial role of Set7/9 in cardiomyocytes (CMs) differentiation and heart development (Fig. 4).

The key role of Set7/9 activity in proper heart morphogenesis was shown in zebrafish models. Firstly, the particularly high expression of Set7/9 in the hearts of 48 h post-fertilization zebrafish embryos was revealed, which indicates the possibility of specific redistribution of the zygotic Set7/9 protein [127]. Furthermore, the knockdown of Set7/9 using specific morpholino caused defective cardiac development and the formation of heart edemas in zebrafish embryos [127]. Interestingly, simultaneous suppression of Set7/9 and SMYD3 methyltransferases, which together can mediate progressive methylation of H3-K4 from mono- to tri-methylated H3K4, caused synergistic defects in heart development, indicating the importance of H3K4 methylation in cardiac formation [127].

The more recent study [114] demonstrated that Set7/9 is a reader of the H3K36Me3 histone methylation mark independently of its enzymatic activity. By recognizing the H3-K36Me3 mark associated with histones at CM differentiation-associated genes, Set7/9 facilitated the recruitment of RNA polymerase II promoting the transcription. It should be emphasized that Set7/9 associates with different co-factors during the particular differentiation stages of CMs. In this respect, Set7/9 was shown to promote the assembly of SWI/SNF chromatin-remodeling complex at the key regulatory genes determining mesoderm and cardiac mesoderm lineages (including MIXL1, DKK, GATA3, and MESP1) during mesoderm commitment. At the later stage, when the cardiac linage specification occurred, Set7/9 was shown to cooperate with the NKX2-5 transcription factor to promote the expression of such important CM differentiation genes as MYH6, GATA4, and ACTC1 [114]. Consistently, the elevation of Set7/9 expression during both hESCs and mESC differentiation towards CMs was also demonstrated [114] (Fig. 4).

### The role of Set7/9 in skeletal muscle formation

In addition to cardiac muscle cells, several reports indicate that Set7/9 participates in the striated muscle formation. Despite the fact that Set7/9 knockout mice do not demonstrate any developmental abnormalities [36, 37, 75, 128], an increasing number of studies point towards the importance of Set7/9 for the formation of appropriate striated muscle fibers during development and regeneration (Fig. 4).

Tao et al. reported that during C2C12 murine myoblast differentiation to myotubes [7] the Set7/9 expression level elevated. Mechanistically, Set7/9 regulates the assembly of myofibrils and the expression of contractive proteins. Accordingly, knockdown of Set7/9 impairs skeletal muscle cell differentiation at the level of gene expression regulation. The lack of Set7/9 prevents the recruitment of myoblast-specific transcription factor MyoD to the regulatory elements of its target genes whose products are involved in skeletal muscle cell differentiation [7]. Importantly, the versality of Set7/9 function through evolutionary-distant groups of organisms was demonstrated: the Set7/9 morpholino injected into 24 h zebrafish embryos caused paralysis, inability to respond to tactile stimuli, impaired sarcomerogenesis, and a dramatic decrease of MHC proteins in striated muscle tissues [7]. Ablation of Set7/9 phenocopied the effect of Suv39h1 since the latter was shown to repress myogenic differentiation [129] via hindering the MyoD transcriptional activity. On the contrary, Set7/9 expression restored the activity of MyoD in part by directly suppressing the Suv39h1 protein [7] (Fig. 4).

Importantly, the activity of Set7/9 itself is apparently regulated during the processes of muscle cell differentiation. Using the C2C12 cell line and the murine amyotrophic lateral sclerosis (ALS) murine model, it was shown that Set7/9 undergoes reversible sumoylation [130]. It was shown that desumoylation of Set7/9 by SENP3 instigates an appropriate sarcomere assembly [130]. Mechanistically, desumoylated Set7/9 was recruited to the myosin heavy chain (MHC) promoter region, where it competed with Suv39h1 to initiate the loading of RNAPolII to promote transcription of the *MhcII* gene. The activity of SENP3 was shown to be perturbed in ALS, resulting in enhanced Set7/9 sumoylation, Suv39h1-mediated repression of *MhcII*, and disorganized sarcomeres in muscle tissues, followed by cachexia [130].

Notably, the same molecular mechanism is involved in anticancer chemotherapy-induced cachexia. In this case, genotoxic drugs daunorubicin and etoposide inhibited SENP3-mediated desumoylation of Set7/9, which in turn impedes the loading of the Set7/9-p300 complex on the *MhcII* promoter. As a result, the Set7/9-dependent cachexia developed in response to genotoxic therapy [131] (Fig. 4).

Another mechanism by which Set7/9 contributes to the myogenic differentiation is exerted via methylation of the serum response transcription factor protein (SRF) [132]. Methylation of SRF at K165 by Set7/9 promotes its binding to SRF response elements (SRE) in the promoter regions of many muscle differentiation-specific genes [132]. Significantly, the role of demethylase KDM2B in elimination of Set7/9-mediated SRF methylation was also demonstrated. KDM2B is a lysine-specific demethylase that is, in particular, known to target H3K4. In line with this, the overexpression of KDM2B in myoblasts resulted in impaired skeletal muscle cell differentiation, and the treatment of C2C12 cells with a selective small molecular inhibitor of Set7/9 methyltransferase activity, (R)-PFI-2, phenocopied the effect of KDM2B [132].

The independently obtained results from Thomas A. Rando and Fabio M. Rossi groups demonstrated the role of Set7/9 in muscle stem cells (MuSC) [133]. According to this study, Set7/9-mediated methylation of β-catenin, promoted its nuclear translocation and subsequent transactivation of several myogenic genes including *Myh1* and *Tgfb3* [133]. Additionally, the (R)-PFI-2 treatment of MuSCs resulted in the retention of β-catenin in the cytoplasm and impaired myogenic differentiation. Interestingly, the (R)-PFI-2-treated hMuSCs were engrafted better into injured muscles of immune deficient mice compared to the non-treated cells [133] (Fig. 4).

The accumulated evidence strongly argues for the important role of Set7/9 in the process of muscle tissue regeneration and hence points to this methyltransferase as a potential therapeutic target for muscle disorders.

### Participation of Set7/9 in differentiation of pancreatic cells

Evidence for the role of Set7/9 in pancreatic development was demonstrated using Xenopus embryos by Franchesca Spagnoli et al. [134] (Fig. 4). The elevated expression of Set7/9 in the anterior endoderm (AE) (the precursor tissue from which the pancreas is formed) was demonstrated at gastrula, neurula, and tadpole stages of *X. laevis* development, as well as in the mouse foregut endoderm and pancreatic rudiments during its embryogenesis. Importantly, the augmented level of Set7/9 expression correlated with the enhanced expression of AE-specific and pancreatic differentiation genes *Foxa2*, *Pdx1*, and *Ptf1a*. Importantly, the overexpression of Set7/9 in the posterior endoderm cells resulted in a redirection of differentiation towards the AE differentiation and the elevated expression of both AE and pancreatic markers. By contrast, Set7/9 MO injection into AE of *X. laevis* embryos caused a strong reduction of AE TFs FOXA2, Prox1, and HNF1b. This was paralleled by the decreased expression of pancreatic genes *Pdx1*, *Ptf1a*, and *Sox9*, which impaired the development of pancreatic buds [134]. Mechanistically, Set7/9 affected histone H3 methylation at the endoderm- and pancreas-specific differentiation genes. Notably, this data is consistent with a more recent study demonstrating the Set7/9 occupancy at promoters of endoderm markers [135].

In concordance with this study, Set7/9 was shown to play an important role in the adult pancreas. Mechanistically, Set7/9 augments the PDX1 protein expression, which is a key transcription factor regulating all stages of pancreatic development and β-cells maturation (reviewed in [136]) (Fig. 4). On the contrary, knockdown of Set7/9 decreased the expression of PDX1 and secretion of insulin [137]. Furthermore, the research by Deering et al. demonstrated that the promoter region of the *Setd7* gene was enriched with the PDX1 protein and its expression was subjected to the PDX1-mediated regulation [138].

A more recent study confirms the effect of Set7/9 both on PDX1 and its transcription targets in mice with conditional knockout of Set7/9 in β-cells [139] (Fig. 4). The Set7/9-mediated methylation of the PDX1 protein at K131, and consequent augmentation of PDX1 transactivation activity promoted the maturation of β-cells [139].

### Participation of Set7/9 in differentiation of other tissues

#### Bone and cartilage

Set7/9 was demonstrated to contribute to the regulation of bone tissue regeneration. Yin et al. showed that the expression of Set7/9 significantly facilitated the process of bone regeneration in rats [140] (Fig. 4). Furthermore, elevated H3K4me3 levels were detected in osteoblasts expressing the Runx2 osteogenic factor. That the depletion Set7/9 on the contrary, inhibited differentiation of human bone marrow stem cells into osteoblasts argues that the Set7/9-Runx2 axis can be an important regulatory mechanism of osteoblast differentiation [140].

In apparent contradiction to the promoting role of Set7/9 in differentiation it was shown that the level of Set7/9 expression decreased during the chondrogenic differentiation of ATDC5 mouse teratocarcinoma cells (Fig. 4). In opposite, knock down of Set7/9 promotes the expression of several chondrocyte markers under hypoxic conditions [77]. The authors suggested that Set7/9 suppressed the chondrogenic differentiation through the Hippo signaling pathway thereby preventing the association of YAP1 and HIF1a at promoter regions of genes involved in chondrogenic differentiation under hypoxic conditions [77].

#### Brown adipose tissue

Son et al. demonstrated the reduction of Set7/9 expression concomitant with sharp activation of H4 histone methyltransferase SUV420H2 during the process of differentiation of immortalized murine brown preadipocytes into mature adipocytes [141]. The follow-up study by the same group revealed that Set7/9 stimulated the expression of UCP-1, PRDM16, and Cidea but did not affect the levels of lipid metabolism regulating factors, PPAR-γ and aP2 [142]. Conversely, knockdown of Set7/9 led to attenuation of thermogenic but not lipid metabolism-regulating factors (Fig. 4). According to this study, Set7/9 acted as a positive regulator of p53 transactivation activity towards thermogenic genes, thus preventing SIRT1-mediated deacetylation and inactivation of p53. Furthermore, Set7/9 knockdown decreased oxygen consumption and the amount of mitochondria of adipocytes, indicating the importance of Set7/9 for the metabolic rate of brown fat cells [142]. It can be speculated that the decrease of Set7/9 levels during the final stage of adipocytes maturation after the elevation of Set7/9 at the earlier stages of differentiation (e.g., MSC determination to adipose lineage) is the physiological mechanism to ensure the execution of the thermogenic genes activation program.

#### Neuronal tissue

High levels of the Set7/9 protein were detected both in the embryonic neural tube of *X. laevis* [134] and in adult murine brain tissue [142] indicating the participation of Set7/9 in differentiation of neuronal cells.

In line with this notion, Fujita et al. demonstrated the importance of Set7/9 in the formation of neural crest-derived stem cells (NCSCs) [143]. Neural crest cells migrating from the embryonic neural fold are precursors of a wide variety of tissue types, including central and peripheral nervous systems, bones and cartilages, smooth muscle tissue, and skin melanocytes [144]. Importantly, some neural crest cells maintain multipotency and are present in such adult tissues as bone marrow, epidermis, cardiac tissue, cornea, and others [145]. Set7/9 was shown to be an important regulator of the expression of transcription factors FoxD3 and Sox10 that are indispensable for the identity and multipotency of NCSCs [146, 147]. Mechanistically, Set7/9 ensures an appropriate level of H3K4 methylation of the FoxD3 enhancer to allow subsequent recruitment of the CHD7 chromatin remodeling factor to activate the transcription [143]. The knockdown of Set7/9 in primary murine embryonic neural crest cells resulted in a reduction of CHD7 binding to the FoxD3 enhancer and consequent suppression of FoxD3 and its transcription target Sox10 expression [143] (Fig. 4).

Furthermore, Set7/9 apparently plays an important role in adult brain cells. Notably, Set7/9 methylates Alzheimer disease (AD)-associated tau protein at K132, facilitating its subsequent methylation at K130 [148]. Importantly, tau methylation is associated with its translocation from neurites to the soma and nuclei of neurons, hyperphosphorylation, accumulation of high-molecular-weight tau forms, and subsequent AD progression [148] (Fig. 4). In accordance, targeting H3K4 methylation with small molecule inhibitors resulted in the recovery of prefrontal cortex synaptic function and memory in AD mouse models and can be considered a promising strategy for AD treatment [149].

Another study demonstrated the role of Set7/9 in the development of postoperative cognitive dysfunction (POCD) caused by anesthesia [150]. Thus, knockdown of Set7/9 suppressed pyroptosis as well as reduced cytokine expression and activation of the NLRP3 inflammasome in the hippocampus of isoflurane-anesthetized mice [150]. Moreover, cognitive characteristics that were compromised by isoflurane anesthesia were improved in Set7/9 knocked-down mice compared to the control group [150] (Fig. 4). Together, these findings indicate the potential role of Set7/9 in age-related brain disorders [151].

#### Mammary glands

The role of Set7/9 was also demonstrated for mammary gland epithelium cells (MEC) [152]. Both Set7/9 mRNA and protein levels increased during differentiation of highly proliferating undifferentiated stem-like cells into differentiated MEC characterized by milk proteins synthesis and lipogenic phenotypes (Fig. 4). Inhibition of Set7/9 methyltransferase activity by (R)-PFI-2 enhanced the proliferation and impaired lactogenic differentiation of murine MEC. It was shown that suppression of Set7/9 attenuated the level of H3K4 methylation in chromatin of various differentiation markers, including E-cadherin, beta-catenin, lactoferrin, insulin-like growth factor binding protein 5, and beta-casein [152]. Additionally, the lipid profile and the expression of phospholipid biosynthesis factors were also altered in Set7/9-suppressed MEC, confirming the importance of Set7/9 in control of differentiation.

## The role of Set7/9 in CSCs

Currently, the concept of CSC has widespread acceptance. CSCs exist as a small population of cancer cells characterized by the expression of such surface markers as CD24, CD44, and/or CD133 [153]. Additionally, the expression of stemness factors Nanog, OCT3/4, SOX2 is largely elevated in CSCs [154]. Different cancer types are shown to express specific biomarker profiles, allowing to distinguish the CSC population from mass of the “normal” non-CSC in a particular malignancy [155–157]. Since CSCs are considered to be responsible for the tumor initiation process, the relapses of the disease, and the development of resistance to various types of antitumor therapy, they remain the primary focus of numerous studies. Several research works demonstrate the participation of Set7/9 in the formation, maintenance, and self-renewal of different types of CSCs.

Thus, a cluster of SC-specific miRNAs miR-372/373 were shown to promote the colorectal CSCs formation, self-renewal, invasive capacity, as well as chemoresistance [158]. Set7/9 mRNA along with some other differentiation factors are targeted by miR-372/373 and downregulated in miR-372/373 expressing colorectal CSCs. In parallel, such colorectal CSCs markers as Nanog, CD24, and CD26 were enhanced in response to miR-372/373 overexpression [158]. Importantly, the level of Set7/9 expression was demonstrated to be significantly higher in normal colon samples compared to malignant samples, which corresponds to the data we demonstrate for Set7/9 levels in breast tumors and non-cancerous breast tissue [159].

In contrast, another study demonstrates that Set7/9, in cooperation with HIF2 and NOTCH1, activates TLR expression in hepatic tumor-initiating stem-like cells (TIC) under hypoxic conditions and hence contributes to the promotion of carcinogenesis and the self-renewal of hepatic TICs [160]. It was also shown that Set7/9 is elevated in TIC compared to CD133^−^ non-CSCs. Interestingly, TLR4, being a target of miRNA let-7, is positively regulated by LIN28, that, as described above, prevents let-7 maturation. Additionally, this study demonstrates that Set7/9 promotes NANOG and CD133 expression through the suppression of DNA methylases DNMT1, DNMT3a, and DNMT3b [160].

It should be noted, that Set7/9-mediated stabilization of LIN28, and, as a consequence, prevention of let-7 maturation [115] may also contribute to BCSCs formation. Indeed, let-7 was shown to suppress BCSCs self-renewal and mammosphere formation, as well as in vivo engraftment and metastasis formation [161].

Since the effect of Set7/9 on CSC-associated molecular pathways is apparently pleiotropic, this warrants their detailed investigation.

## Conclusion

In this review, we summarized the information available to date on Set7/9 participation in vital but opposite biological processes: cellular differentiation and embryogenesis. One important conclusion that emerges from the systematic literature review on Set7/9 is that the latter can facilitate either differentiation or self-renewal, depending on the cellular context. Given that both gene expression programs are ignited by specific transcription factors that in turn recruit particular sets of histone modifications, it can be assumed that Set7/9 being both histone- and non-histone-specific methyltransferase is involved at multiple levels.

In line with notion is the fact that although the majority of reports and our own data indicate that Set7/9 primarily acts as a pro-differentiation factor that contributes to SC specification, there are some studies demonstrating that Set7/9 exerts stemness-promoting effects, indicating the multifaceted and context-dependent role of this methyltransferase in development and differentiation. These reports warrant a closer look at the assortment of pioneering transcription factors in each particular case because this may define the biological outcome of Set7/9 activity.

Not surprisingly, Set7/9 is involved in multiple pathological processes such as aberrant development, inappropriate tissue regeneration and functioning, neurodegeneration, development of resistance to therapy, and cancer relapse. Thus, on the basis of the data summarized here, modulation of Set7/9 activity may be considered a promising strategy for targeted therapy of various pathologies.

## References

[1] Yang C, Zhang J, Ma Y, Wu C, Cui W, Wang L (2020). Histone methyltransferase and drug resistance in cancers. J Exp Clin Cancer Res.

[2] Lazar T, Schad E, Szabo B, Horvath T, Meszaros A, Tompa P, Tantos A (2016). Intrinsic protein disorder in histone lysine methylation. Biol Direct.

[3] Erlendson AA, Freitag M (2022). Not all is SET for methylation: evolution of eukaryotic protein methyltransferases. Histone methyltransferases: methods and protocols.

[4] Freitag M (2017). Histone methylation by SET domain proteins in fungi. Annu Rev Microbiol.

[5] Nishioka K, Chuikov S, Sarma K, Erdjument-Bromage H, Allis CD, Tempst P, Reinberg D (2002). Set9, a novel histone H3 methyltransferase that facilitates transcription by precluding histone tail modifications required for heterochromatin formation. Genes Dev.

[6] Wang H, Cao R, Xia L, Erdjument-Bromage H, Borchers C, Tempst P, Zhang Y (2001). Purification and functional characterization of a histone H3-lysine 4-specific methyltransferase. Mol Cell.

[7] Tao Y, Neppl RL, Huang Z-P, Chen J, Tang R-H, Cao R (2011). The histone methyltransferase Set7/9 promotes myoblast differentiation and myofibril assembly. J Cell Biol.

[8] Chuikov S, Kurash JK, Wilson JR, Xiao B, Justin N, Ivanov GS (2004). Regulation of p53 activity through lysine methylation. Nature.

[9] Daks A, Shuvalov O, Fedorova O, Parfenyev S, Simon H-U, Barlev NA (2023). Methyltransferase set7/9 as a multifaceted regulator of ROS response. Int J Biol Sci.

[10] Daks A, Vasileva E, Fedorova O, Shuvalov O, Barlev NA (2022). The role of lysine methyltransferase SET7/9 in proliferation and cell stress response. Life.

[11] Ivanov GS, Ivanova T, Kurash J, Ivanov A, Chuikov S, Gizatullin F (2007). Methylation-acetylation interplay activates p53 in response to DNA damage. Mol Cell Biol.

[12] Vasileva E, Shuvalov O, Petukhov A, Fedorova O, Daks A, Nader R, Barlev N (2020). KMT Set7/9 is a new regulator of Sam68 STAR-protein. Biochem Biophys Res Commun.

[13] Collins BE, Greer CB, Coleman BC, Sweatt JD (2019). Histone H3 lysine K4 methylation and its role in learning and memory. Epigenet Chromatin.

[14] Morgunkova A, Barlev NA (2006). Lysine methylation goes global. Cell Cycle.

[15] Nightingale KP, Gendreizig S, White DA, Bradbury C, Hollfelder F, Turner BM (2007). Cross-talk between histone modifications in response to histone deacetylase inhibitors: MLL4 links histone H3 acetylation and histone H3K4 methylation. J Biol Chem.

[16] Zhang K, Siino JS, Jones PR, Yau PM, Bradbury EM (2004). A mass spectrometric “Western blot” to evaluate the correlations between histone methylation and histone acetylation. Proteomics.

[17] Mattout A, Biran A, Meshorer E (2011). Global epigenetic changes during somatic cell reprogramming to iPS cells. J Mol Cell Biol.

[18] Mikkelsen TS, Hanna J, Zhang X, Ku M, Wernig M, Schorderet P (2008). Dissecting direct reprogramming through integrative genomic analysis. Nature.

[19] Ang Y-S, Tsai S-Y, Lee D-F, Monk J, Su J, Ratnakumar K (2011). Wdr5 mediates self-renewal and reprogramming via the embryonic stem cell core transcriptional network. Cell.

[20] Macrae TA, Fothergill-Robinson J, Ramalho-Santos M (2023). Regulation, functions and transmission of bivalent chromatin during mammalian development. Nat Rev Mol Cell Biol.

[21] Blanco E, González-Ramírez M, Alcaine-Colet A, Aranda S, Di Croce L (2020). The bivalent genome: characterization, structure, and regulation. Trends Genet.

[22] Matsumura Y, Nakaki R, Inagaki T, Yoshida A, Kano Y, Kimura H (2015). H3K4/H3K9me3 bivalent chromatin domains targeted by lineage-specific DNA methylation pauses adipocyte differentiation. Mol Cell.

[23] Bernstein BE, Mikkelsen TS, Xie X, Kamal M, Huebert DJ, Cuff J (2006). A bivalent chromatin structure marks key developmental genes in embryonic stem cells. Cell.

[24] Sze CC, Cao K, Collings CK, Marshall SA, Rendleman EJ, Ozark PA (2017). Histone H3K4 methylation-dependent and-independent functions of Set1A/COMPASS in embryonic stem cell self-renewal and differentiation. Genes Dev.

[25] Fang L, Zhang J, Zhang H, Yang X, Jin X, Zhang L (2016). H3K4 methyltransferase Set1a is a key Oct4 coactivator essential for generation of Oct4 positive inner cell mass. Stem Cells.

[26] Bledau AS, Schmidt K, Neumann K, Hill U, Ciotta G, Gupta A (2014). The H3K4 methyltransferase Setd1a is first required at the epiblast stage, whereas Setd1b becomes essential after gastrulation. Development.

[27] Ardehali MB, Mei A, Zobeck KL, Caron M, Lis JT, Kusch T (2011). Drosophila Set1 is the major histone H3 lysine 4 trimethyltransferase with role in transcription. EMBO J.

[28] Wu M, Wang PF, Lee JS, Martin-Brown S, Florens L, Washburn M, Shilatifard A (2008). Molecular regulation of H3K4 trimethylation by Wdr82, a component of human Set1/COMPASS. Mol Cell Biol.

[29] Chakrabarti SK, Francis J, Ziesmann SM, Garmey JC, Mirmira RG (2003). Covalent histone modifications underlie the developmental regulation of insulin gene transcription in pancreatic β cells. J Biol Chem.

[30] Wang Z, Ren B (2024). Role of H3K4 monomethylation in gene regulation. Curr Opin Genet Dev.

[31] Yan J, Chen SAA, Local A, Liu T, Qiu Y, Dorighi KM (2018). Histone H3 lysine 4 monomethylation modulates long-range chromatin interactions at enhancers. Cell Res.

[32] Heintzman ND, Stuart RK, Hon G, Fu Y, Ching CW, Hawkins RD (2007). Distinct and predictive chromatin signatures of transcriptional promoters and enhancers in the human genome. Nat Genet.

[33] Dhayalan A, Kudithipudi S, Rathert P, Jeltsch A (2011). Specificity analysis-based identification of new methylation targets of the SET7/9 protein lysine methyltransferase. Chem Biol.

[34] Mattiroli F, Vissers JH, van Dijk WJ, Ikpa P, Citterio E, Vermeulen W (2012). RNF168 ubiquitinates K13–15 on H2A/H2AX to drive DNA damage signaling. Cell.

[35] Sekiguchi M, Matsushita N (2022). DNA damage response regulation by histone ubiquitination. Int J Mol Sci.

[36] Campaner S, Spreafico F, Burgold T, Doni M, Rosato U, Amati B, Testa G (2011). The methyltransferase Set7/9 (Setd7) is dispensable for the p53-mediated DNA damage response in vivo. Mol Cell.

[37] Lehnertz B, Rogalski JC, Schulze FM, Yi L, Lin S, Kast J, Rossi FM (2011). p53-dependent transcription and tumor suppression are not affected in Set7/9-deficient mice. Mol Cell.

[38] Barth TK, Imhof A (2010). Fast signals and slow marks: the dynamics of histone modifications. Trends Biochem Sci.

[39] Castaño J, Morera C, Sesé B, Boue S, Bonet-Costa C, Martí M (2016). SETD7 regulates the differentiation of human embryonic stem cells. PLoS ONE.

[40] Prendergast L, Reinberg D (2021). The missing linker: emerging trends for H1 variant-specific functions. Genes Dev.

[41] Wang D, Zhou J, Liu X, Lu D, Shen C, Du Y (2013). Methylation of SUV39H1 by SET7/9 results in heterochromatin relaxation and genome instability. Proc Natl Acad Sci.

[42] Sun H, Wang Y, Wang Y, Ji F, Wang A, Yang M (2022). Bivalent regulation and related mechanisms of H3K4/27/9me3 in stem cells. Stem Cell Rev Rep.

[43] Scarola M, Comisso E, Pascolo R, Chiaradia R, Maria Marion R, Schneider C (2015). Epigenetic silencing of Oct4 by a complex containing SUV39H1 and Oct4 pseudogene lncRNA. Nat Commun.

[44] Guerra MV, Cáceres MI, Herrera-Soto A, Arredondo SB, Varas-Godoy M, van Zundert B, Varela-Nallar L (2022). H3K9 methyltransferases Suv39h1 and Suv39h2 control the differentiation of neural progenitor cells in the adult hippocampus. Front Cell Dev Biol.

[45] Pace L, Goudot C, Zueva E, Gueguen P, Burgdorf N, Waterfall JJ (2018). The epigenetic control of stemness in CD8+ T cell fate commitment. Science.

[46] Tsai C-C, Su P-F, Huang Y-F, Yew T-L, Hung S-C (2012). Oct4 and Nanog directly regulate Dnmt1 to maintain self-renewal and undifferentiated state in mesenchymal stem cells. Mol Cell.

[47] Trowbridge JJ, Snow JW, Kim J, Orkin SH (2009). DNA methyltransferase 1 is essential for and uniquely regulates hematopoietic stem and progenitor cells. Cell Stem Cell.

[48] Goto K, Numata M, Komura J-I, Ono T, Bestor TH, Kondo H (1994). Expression of DNA methyltransferase gene in mature and immature neurons as well as proliferating cells in mice. Differentiation.

[49] Noguchi H, Murao N, Kimura A, Matsuda T, Namihira M, Nakashima K (2016). DNA methyltransferase 1 is indispensable for development of the hippocampal dentate gyrus. J Neurosci.

[50] Sen GL, Reuter JA, Webster DE, Zhu L, Khavari PA (2010). DNMT1 maintains progenitor function in self-renewing somatic tissue. Nature.

[51] Li E, Bestor TH, Jaenisch R (1992). Targeted mutation of the DNA methyltransferase gene results in embryonic lethality. Cell.

[52] Wang J, Hevi S, Kurash JK, Lei H, Gay F, Bajko J (2009). The lysine demethylase LSD1 (KDM1) is required for maintenance of global DNA methylation. Nat Genet.

[53] Estève P-O, Chin HG, Benner J, Feehery GR, Samaranayake M, Horwitz GA (2009). Regulation of DNMT1 stability through SET7-mediated lysine methylation in mammalian cells. Proc Natl Acad Sci.

[54] Amelio I, Mancini M, Petrova V, Cairns RA, Vikhreva P, Nicolai S (2018). p53 mutants cooperate with HIF-1 in transcriptional regulation of extracellular matrix components to promote tumor progression. Proc Natl Acad Sci.

[55] Kim Y, Nam HJ, Lee J, Kim C, Yu YS, Kim D (2016). Methylation-dependent regulation of HIF-1α stability restricts retinal and tumour angiogenesis. Nat Commun.

[56] Fuellen G, Struckmann S (2010). Evolution of gene regulation of pluripotency-the case for wiki tracks at genome browsers. Biol Direct.

[57] Yang Z, Liu Z, Lu W, Guo H, Chen J, Zhang Y (2023). LncRNA WAC-AS1 promotes osteosarcoma Metastasis and stemness by sponging miR-5047 to upregulate SOX2. Biol Direct.

[58] Holloway DT, Kon M, DeLisi C (2008). In silico regulatory analysis for exploring human disease progression. Biol Direct.

[59] Heng J-CD, Ng H-H (2010). Transcriptional regulation in embryonic stem cells. Cell Biol Stem Cells.

[60] Young RA (2011). Control of the embryonic stem cell state. Cell.

[61] Fang L, Zhang L, Wei W, Jin X, Wang P, Tong Y (2014). A methylation-phosphorylation switch determines Sox2 stability and function in ESC maintenance or differentiation. Mol Cell.

[62] Zhang C, Hoang N, Leng F, Saxena L, Lee L, Alejo S (2018). LSD1 demethylase and the methyl-binding protein PHF20L1 prevent SET7 methyltransferase–dependent proteolysis of the stem-cell protein SOX2. J Biol Chem.

[63] Kühl SJ, Kühl M (2013). On the role of Wnt/β-catenin signaling in stem cells. Biochim Biophys Acta BBA Gen Subj.

[64] Melino G, Memmi EM, Pelicci PG, Bernassola F (2015). Maintaining epithelial stemness with p63. Sci Signal.

[65] Sun J (2011). Enteric bacteria and cancer stem cells. Cancers.

[66] Moon RT, Bowerman B, Boutros M, Perrimon N (2002). The promise and perils of Wnt signaling through β-catenin. Science.

[67] Huang Y, Wan S, Yang M (2021). Circ_0067680 expedites the osteogenic differentiation of human bone marrow-derived mesenchymal stem cells through miR-4429/CTNNB1/Wnt/β-catenin pathway. Biol Direct.

[68] Heng BC, Zhang X, Aubel D, Bai Y, Li X, Wei Y (2020). Role of YAP/TAZ in cell lineage fate determination and related signaling pathways. Front Cell Dev Biol.

[69] Driskill JH, Pan D (2023). Control of stem cell renewal and fate by YAP and TAZ. Nat Rev Mol Cell Biol.

[70] Monroe TO, Hill MC, Morikawa Y, Leach JP, Heallen T, Cao S (2019). YAP partially reprograms chromatin accessibility to directly induce adult cardiogenesis in vivo. Dev Cell.

[71] Varelas X, Sakuma R, Samavarchi-Tehrani P, Peerani R, Rao BM, Dembowy J (2008). TAZ controls Smad nucleocytoplasmic shuttling and regulates human embryonic stem-cell self-renewal. Nat Cell Biol.

[72] Bora-Singhal N, Nguyen J, Schaal C, Perumal D, Singh S, Coppola D, Chellappan S (2015). YAP1 regulates OCT4 activity and SOX2 expression to facilitate self-renewal and vascular mimicry of stem-like cells. Stem cells.

[73] Shen C, Wang D, Liu X, Gu B, Du Y, Wei FZ (2015). SET7/9 regulates cancer cell proliferation by influencing β-catenin stability. FASEB J.

[74] Wang D, Li Y, Xu C, Wang H, Huang X, Jin X (2023). SETD7 promotes lateral plate mesoderm formation by modulating the Wnt/β-catenin signaling pathway. Iscience.

[75] Oudhoff MJ, Freeman SA, Couzens AL, Antignano F, Kuznetsova E, Min PH (2013). Control of the hippo pathway by Set7-dependent methylation of Yap. Dev Cell.

[76] Barsyte-Lovejoy D, Li F, Oudhoff MJ, Tatlock JH, Dong A, Zeng H (2014). (R)-PFI-2 is a potent and selective inhibitor of SETD7 methyltransferase activity in cells. Proc Natl Acad Sci.

[77] Li M, Ning J, Wang J, Yan Q, Zhao K, Jia X (2021). SETD7 regulates chondrocyte differentiation and glycolysis via the Hippo signaling pathway and HIF-1α. Int J Mol Med.

[78] Ambrosini S, Montecucco F, Kolijn D, Pedicino D, Akhmedov A, Mohammed SA (2022). Methylation of the Hippo effector YAP by the methyltransferase SETD7 drives myocardial ischaemic injury: a translational study. Cardiovasc Res.

[79] Ambrosini S, Montecucco F, Akhmedov A, Mohammed S, Brown P, Rossi F (2020). Methylation of the hippo signalling effector YAP by SETD7 drives myocardial ischemic injury. Eur Heart J.

[80] Chen S, Wu H, Wang Z, Jia M, Guo J, Jin J (2022). Loss of SPTBN1 suppresses autophagy via SETD7-mediated YAP methylation in hepatocellular carcinoma initiation and development. Cell Mol Gastroenterol Hepatol.

[81] Oudhoff MJ, Braam MJ, Freeman SA, Wong D, Rattray DG, Wang J (2016). SETD7 controls intestinal regeneration and tumorigenesis by regulating Wnt/β-catenin and Hippo/YAP signaling. Dev Cell.

[82] Heo J, Lim J, Lee S, Jeong J, Kang H, Kim Y (2017). Sirt1 regulates DNA methylation and differentiation potential of embryonic stem cells by antagonizing Dnmt3l. Cell Rep.

[83] Zainabadi K (2018). The variable role of SIRT1 in the maintenance and differentiation of mesenchymal stem cells. Regen Med.

[84] Simic P, Zainabadi K, Bell E, Sykes DB, Saez B, Lotinun S (2013). SIRT1 regulates differentiation of mesenchymal stem cells by deacetylating β-catenin. EMBO Mol Med.

[85] Chen H, Liu X, Chen H, Cao J, Zhang L, Hu X, Wang J (2014). Role of SIRT1 and AMPK in mesenchymal stem cells differentiation. Ageing Res Rev.

[86] Wang Y, Tian C, Zheng JC (2013). FoxO3a contributes to the reprogramming process and the differentiation of induced pluripotent stem cells. Stem Cells Dev.

[87] Bigarella CL, Li J, Rimmelé P, Liang R, Sobol RW, Ghaffari S (2017). FOXO3 transcription factor is essential for protecting hematopoietic stem and progenitor cells from oxidative DNA damage. J Biol Chem.

[88] Gopinath SD, Webb AE, Brunet A, Rando TA (2014). FOXO3 promotes quiescence in adult muscle stem cells during the process of self-renewal. Stem Cell Rep.

[89] Yeo H, Lyssiotis CA, Zhang Y, Ying H, Asara JM, Cantley LC, Paik JH (2013). FoxO3 coordinates metabolic pathways to maintain redox balance in neural stem cells. EMBO J.

[90] Yang X, Wang Y, Rovella V, Candi E, Jia W, Bernassola F (2023). Aged mesenchymal stem cells and inflammation: from pathology to potential therapeutic strategies. Biol Direct.

[91] Sun W, Qiao W, Zhou B, Hu Z, Yan Q, Wu J (2018). Overexpression of Sirt1 in mesenchymal stem cells protects against bone loss in mice by FOXO3a deacetylation and oxidative stress inhibition. Metabolism.

[92] Xie Q, Hao Y, Tao L, Peng S, Rao C, Chen H (2012). Lysine methylation of FOXO3 regulates oxidative stress-induced neuronal cell death. EMBO Rep.

[93] Calnan DR, Webb AE, White JL, Stowe TR, Goswami T, Shi X (2012). Methylation by Set9 modulates FoxO3 stability and transcriptional activity. Aging (Albany NY).

[94] Liu X, Wang D, Zhao Y, Tu B, Zheng Z, Wang L (2011). Methyltransferase Set7/9 regulates p53 activity by interacting with Sirtuin 1 (SIRT1). Proc Natl Acad Sci.

[95] Takahashi K, Yamanaka S (2006). Induction of pluripotent stem cells from mouse embryonic and adult fibroblast cultures by defined factors. Cell.

[96] Arnold I, Watt FM (2001). c-Myc activation in transgenic mouse epidermis results in mobilization of stem cells and differentiation of their progeny. Curr Biol.

[97] Sumi T, Tsuneyoshi N, Nakatsuji N, Suemori H (2007). Apoptosis and differentiation of human embryonic stem cells induced by sustained activation of c-Myc. Oncogene.

[98] Varlakhanova NV, Cotterman RF, deVries WN, Morgan J, Donahue LR, Murray S (2010). Myc maintains embryonic stem cell pluripotency and self-renewal. Differentiation.

[99] Lin CH, Jackson AL, Guo J, Linsley PS, Eisenman RN (2009). Myc-regulated microRNAs attenuate embryonic stem cell differentiation. EMBO J.

[100] Yoshida GJ (2018). Emerging roles of Myc in stem cell biology and novel tumor therapies. J Exp Clin Cancer Res.

[101] Rodriguez R, Rubio R, Masip M, Catalina P, Nieto A, DeCueva T (2009). Loss of p53 induces tumorigenesis in p21-deficient mesenchymal stem cells. Neoplasia.

[102] Zheng H, Ying H, Yan H, Kimmelman AC, Hiller DJ, Chen A-J (2008). p53 and Pten control neural and glioma stem/progenitor cell renewal and differentiation. Nature.

[103] Akita H, Marquardt JU, Durkin ME, Kitade M, Seo D, Conner EA (2014). MYC activates stem-like cell potential in hepatocarcinoma by a p53-dependent mechanism. Can Res.

[104] Lezina L, Aksenova V, Fedorova O, Malikova D, Shuvalov O, Antonov AV (2015). KMT Set7/9 affects genotoxic stress response via the Mdm2 axis. Oncotarget.

[105] Daks A, Shuvalov O, Fedorova O, Petukhov A, Lezina L, Zharova A (2021). p53-independent effects of Set7/9 lysine methyltransferase on metabolism of non-small cell lung cancer cells. Front Oncol.

[106] Ito K, Suda T (2014). Metabolic requirements for the maintenance of self-renewing stem cells. Nat Rev Mol Cell Biol.

[107] Batista IdAA, Helguero LA (2018). Biological processes and signal transduction pathways regulated by the protein methyltransferase SETD7 and their significance in cancer. Signal Transduct Target Therapy.

[108] Chiang C, Yang H, Zhu L, Chen C, Zheng D (2022). The epigenetic regulation of nonhistone proteins by SETD7: new targets in cancer. Front Genet.

[109] Jiang B-H, Tseng W-L, Li H-Y, Wang M-L, Chang Y-L, Sung Y-J, Chiou S-H (2015). Poly (ADP-ribose) polymerase 1: cellular pluripotency, reprogramming, and tumorogenesis. Int J Mol Sci.

[110] Xie D, Pei Q, Li J, Wan X, Ye T (2021). Emerging role of E2F family in cancer stem cells. Front Oncol.

[111] Kaltschmidt C, Banz-Jansen C, Benhidjeb T, Beshay M, Förster C, Greiner J (2019). A role for NF-κB in organ specific cancer and cancer stem cells. Cancers.

[112] Kaltschmidt C, Greiner JF, Kaltschmidt B (2021). The transcription factor NF-κB in stem cells and development. Cells.

[113] Huang X, Trinh T, Aljoufi A, Broxmeyer HE (2018). Hypoxia signaling pathway in stem cell regulation: good and evil. Curr Stem Cell Rep.

[114] Lee J, Shao N-Y, Paik DT, Wu H, Guo H, Termglinchan V (2018). SETD7 drives cardiac lineage commitment through stage-specific transcriptional activation. Cell Stem Cell.

[115] Kim S-K, Lee H, Han K, Kim SC, Choi Y, Park S-W (2014). SET7/9 methylation of the pluripotency factor LIN28A is a nucleolar localization mechanism that blocks let-7 biogenesis in human ESCs. Cell Stem Cell.

[116] Divisato G, Passaro F, Russo T, Parisi S (2020). The key role of microRNAs in self-renewal and differentiation of embryonic stem cells. Int J Mol Sci.

[117] Martinez NJ, Gregory RI (2010). MicroRNA gene regulatory pathways in the establishment and maintenance of ESC identity. Cell Stem Cell.

[118] Stadler B, Ivanovska I, Mehta K, Song S, Nelson A, Tan Y (2010). Characterization of microRNAs involved in embryonic stem cell states. Stem Cells Dev.

[119] Martin-Lopez M, Maeso-Alonso L, Fuertes-Alvarez S, Balboa D, Rodríguez-Cortez V, Weltner J (2017). p73 is required for appropriate BMP-induced mesenchymal-to-epithelial transition during somatic cell reprogramming. Cell Death Dis.

[120] Chang C-J, Chao C-H, Xia W, Yang J-Y, Xiong Y, Li C-W (2011). p53 regulates epithelial–mesenchymal transition and stem cell properties through modulating miRNAs. Nat Cell Biol.

[121] Kim T, Veronese A, Pichiorri F, Lee TJ, Jeon Y-J, Volinia S (2011). p53 regulates epithelial–mesenchymal transition through microRNAs targeting ZEB1 and ZEB2. J Exp Med.

[122] Parfenyev S, Singh A, Fedorova O, Daks A, Kulshreshtha R, Barlev NA (2021). Interplay between p53 and non-coding RNAs in the regulation of EMT in breast cancer. Cell Death Dis.

[123] Montenegro M, Sánchez-Del-Campo L, González-Guerrero R, Martínez-Barba E, Piñero-Madrona A, Cabezas-Herrera J, Rodríguez-López J (2016). Tumor suppressor SET9 guides the epigenetic plasticity of breast cancer cells and serves as an early-stage biomarker for predicting metastasis. Oncogene.

[124] Monteiro FL, Williams C, Helguero LA (2022). A systematic review to define the multi-faceted role of lysine methyltransferase SETD7 in cancer. Cancers.

[125] Rozenberg JM, Zvereva S, Dalina A, Blatov I, Zubarev I, Luppov D (2021). The p53 family member p73 in the regulation of cell stress response. Biol Direct.

[126] Goyal A, Chavez SL, Reijo Pera RA (2013). Generation of human induced pluripotent stem cells using epigenetic regulators reveals a germ cell-like identity in partially reprogrammed colonies. PLoS ONE.

[127] Kim J-D, Kim E, Koun S, Ham H-J, Rhee M, Kim M-J, Huh T-L (2015). Proper activity of histone H3 lysine 4 (H3K4) methyltransferase is required for morphogenesis during zebrafish cardiogenesis. Mol Cells.

[128] Elkouris M, Kontaki H, Stavropoulos A, Antonoglou A, Nikolaou KC, Samiotaki M (2016). SET9-mediated regulation of TGF-β signaling links protein methylation to pulmonary fibrosis. Cell Rep.

[129] Mal AK (2006). Histone methyltransferase Suv39h1 represses MyoD-stimulated myogenic differentiation. EMBO J.

[130] Nayak A, Lopez-Davila AJ, Kefalakes E, Holler T, Kraft T, Amrute-Nayak M (2019). Regulation of SETD7 methyltransferase by SENP3 is crucial for sarcomere organization and cachexia. Cell Rep.

[131] Amrute-Nayak M, Pegoli G, Holler T, Lopez-Davila AJ, Lanzuolo C, Nayak A (2021). Chemotherapy triggers cachexia by deregulating synergetic function of histone-modifying enzymes. J Cachexia Sarcopenia Muscle.

[132] Kwon D-H, Kang J-Y, Joung H, Kim J-Y, Jeong A, Min H-K (2021). SRF is a nonhistone methylation target of KDM2B and SET7 in the regulation of skeletal muscle differentiation. Exp Mol Med.

[133] Judson RN, Quarta M, Oudhoff MJ, Soliman H, Yi L, Chang CK (2018). Inhibition of methyltransferase Setd7 allows the in vitro expansion of myogenic stem cells with improved therapeutic potential. Cell Stem Cell.

[134] Kofent J, Zhang J, Spagnoli FM (2016). The histone methyltransferase Setd7 promotes pancreatic progenitor identity. Development.

[135] Kartikasari AE, Zhou JX, Kanji MS, Chan DN, Sinha A, Grapin-Botton A (2013). The histone demethylase Jmjd3 sequentially associates with the transcription factors Tbx3 and Eomes to drive endoderm differentiation. EMBO J.

[136] Ebrahim N, Shakirova K, Dashinimaev E (2022). PDX1 is the cornerstone of pancreatic β-cell functions and identity. Front Mol Biosci.

[137] Jetton TL, Flores-Bringas P, Leahy JL, Gupta D (2021). SetD7 (Set7/9) is a novel target of PPARγ that promotes the adaptive pancreatic β-cell glycemic response. J Biological Chem.

[138] Deering TG, Ogihara T, Trace AP, Maier B, Mirmira RG (2009). Methyltransferase Set7/9 maintains transcription and euchromatin structure at islet-enriched genes. Diabetes.

[139] Maganti AV, Maier B, Tersey SA, Sampley ML, Mosley AL, Özcan S (2015). Transcriptional activity of the islet β cell factor Pdx1 is augmented by lysine methylation catalyzed by the methyltransferase Set7/9. J Biol Chem.

[140] Yin C, Jia X, Miron RJ, Long Q, Xu H, Wei Y (2018). Setd7 and its contribution to Boron-induced bone regeneration in Boron-mesoporous bioactive glass scaffolds. Acta Biomater.

[141] Son MJ, Kim WK, Oh K-J, Park A, Han BS, Lee SC, Bae K-H (2016). Methyltransferase and demethylase profiling studies during brown adipocyte differentiation. BMB Rep.

[142] Son MJ, Kim WK, Park A, Oh K-J, Kim J-H, Han BS (2016). Set7/9, a methyltransferase, regulates the thermogenic program during brown adipocyte differentiation through the modulation of p53 acetylation. Mol Cell Endocrinol.

[143] Fujita K, Ogawa R, Ito K (2016). CHD7, Oct3/4, Sox2, and Nanog control FoxD3 expression during mouse neural crest-derived stem cell formation. FEBS J.

[144] Kunisada T, Tezulka KI, Aoki H, Motohashi T (2014). The stemness of neural crest cells and their derivatives. Birth Defects Res C Embryo Today.

[145] Achilleos A, Trainor PA (2012). Neural crest stem cells: discovery, properties and potential for therapy. Cell Res.

[146] Sauka-Spengler T, Bronner-Fraser M (2008). Evolution of the neural crest viewed from a gene regulatory perspective. Genesis.

[147] Meulemans D, Bronner-Fraser M (2004). Gene-regulatory interactions in neural crest evolution and development. Dev Cell.

[148] Bichmann M, Prat Oriol N, Ercan-Herbst E, Schöndorf DC, Gomez Ramos B, Schwärzler V (2021). SETD7-mediated monomethylation is enriched on soluble Tau in Alzheimer’s disease. Mol Neurodegener.

[149] Cao Q, Wang W, Williams JB, Yang F, Wang Z-J, Yan Z (2020). Targeting histone K4 trimethylation for treatment of cognitive and synaptic deficits in mouse models of Alzheimer’s disease. Sci Adv.

[150] Ma C, Yu X, Li D, Fan Y, Tao Q, Tang Y, Zheng L (2022). Inhibition of SET domain–containing (lysine methyltransferase) 7 alleviates cognitive impairment through suppressing the activation of NOD-like receptor protein 3 inflammasome in isoflurane-induced aged mice. Hum Exp Toxicol.

[151] Han Y, Han D, Yan Z, Boyd-Kirkup JD, Green CD, Khaitovich P, Han JDJ (2012). Stress-associated H 3 K 4 methylation accumulates during postnatal development and aging of rhesus macaque brain. Aging Cell.

[152] Monteiro FL, Góis A, Direito I, Melo T, Neves B, Alves MI (2023). Inhibiting SETD7 methyl-transferase activity impairs differentiation, lipid metabolism and lactogenesis in mammary epithelial cells. FEBS Lett.

[153] Yu Z, Pestell TG, Lisanti MP, Pestell RG (2012). Cancer stem cells. Int J Biochem Cell Biol.

[154] Walcher L, Kistenmacher A-K, Suo H, Blaudszun A-R, Yevsa T, Kossatz-Boehlert U (2020). Cancer stem cells—origins and biomarkers: perspectives for targeted personalized therapies. Front Immunol.

[155] Peired AJ, Sisti A, Romagnani P (2016). Renal cancer stem cells: characterization and targeted therapies. Stem Cells Int..

[156] Lee TK-W, Guan X-Y, Ma S (2022). Cancer stem cells in hepatocellular carcinoma—from origin to clinical implications. Nat Rev Gastroenterol Hepatol.

[157] Gzil A, Zarębska I, Bursiewicz W, Antosik P, Grzanka D, Szylberg Ł (2019). Markers of pancreatic cancer stem cells and their clinical and therapeutic implications. Mol Biol Rep.

[158] Wang LQ, Yu P, Li B, Guo YH, Liang ZR, Zheng LL (2018). miR-372 and miR-373 enhance the stemness of colorectal cancer cells by repressing differentiation signaling pathways. Mol Oncol.

[159] Myadelets D, Parfenyev S, Vasileva J, Shuvalov O, Petukhov A, Fedorova O (2024). Methyltransferase Set7/9 controls PARP1 expression and regulates cisplatin response of breast cancer cells. Biochem Biophys Res Commun.

[160] Hernandez JC, Chen C-L, Machida T, Kumar DBU, Tahara SM, Montana J (2023). LIN28 and histone H3K4 methylase induce TLR4 to generate tumor-initiating stem-like cells. Iscience.

[161] Yu F, Yao H, Zhu P, Zhang X, Pan Q, Gong C (2007). let-7 regulates self renewal and tumorigenicity of breast cancer cells. Cell.

